# Associations between perceptions and sick leave duration in dyads of workers and significant others

**DOI:** 10.1093/eurpub/ckac129.314

**Published:** 2022-10-25

**Authors:** NC Snippen, HJ de Vries, CAM Roelen, M Hagedoorn, S Brouwer

**Affiliations:** 1 Department of Health Sciences, University Medical Center Groningen, Groningen, Netherlands; 2 Arbo Unie, Nieuwegein, Netherlands

## Abstract

**Background:**

Dyadic processes of workers and significant others like partners, family members and friends can play an important role in adaptation to chronic disease, thereby influencing health and work outcomes. This study aimed to increase our understanding of dyadic processes in sick leave duration of workers with chronic diseases. We examined illness perceptions, return to work expectations (RTWE) and perceptions about significant other responses (i.e., engagement, buffering and overprotection) of workers and their significant others in relation to sick leave duration.

**Methods:**

This study used survey and registry data of 90 dyads of sick-listed workers with a chronic disease and their significant others. Simple and multiple regressions in which perceptions of workers and significant others were included simultaneously were used to examine associations with sick leave duration.

**Results:**

Workers’ and significant others’ perceptions were moderately to strongly correlated (r ranged from .46 to .80). Sick leave duration was associated with illness perceptions of both workers (b = 8.58, p=.001) and significant others (b = 6.46, p=.008), with more negative illness perceptions associated with a longer sickness absence. In the multiple regression, illness perceptions explained 12.3% of the variation in sick leave duration. Sick leave duration was also associated with RTWE of workers (b=-76.87, p<.001) and their significant others (b=-92.47, p<.001), with more positive RTWE associated with a shorter sickness absence. The RTWE of dyad members accounted for 24.5% of the variance of sick leave duration.

**Conclusions:**

Illness perceptions and RTWE of workers and their significant others are strongly interdependent and associated with sick leave duration of workers with chronic diseases. A dyadic approach targeted at improving illness perceptions and RTWE of both workers and significant others might be more effective than an individualistic approach in preventing long-term sickness absence.

**Key messages:**

• Return to work expectations and illness perceptions of workers and their significant others are associated with the duration of sickness absence of workers with chronic diseases.

• A dyadic approach targeted at both workers and their significant others might be more effective than an individualistic approach in the prevention of long-term sickness absence.

